# Genetic Variation in Ornamental and Growth Traits in Hybrid Populations of *Lilium davidii* var. *unicolor*

**DOI:** 10.3390/plants14050656

**Published:** 2025-02-21

**Authors:** Yufei Han, Pengcheng Yu, Yuzhou Jiang, Ningya Chen, Tiangeng Gong, Xiangfeng Kong, Li Gao, Guixia Jia

**Affiliations:** 1 Beijing Key Laboratory of Ornamental Plants Germplasm Innovation & Molecular Breeding, National Engineering Research Center for Floriculture, Beijing Laboratory of Urban and Rural Ecological Environment, Key Laboratory of Genetics and Breeding in Forest Trees and Ornamental Plants of Ministry of Education, School of Landscape Architecture, Beijing Forestry University, Beijing 100083, China; 2 Beijing Green Garden Group Co., Ltd., Beijing 100067, China

**Keywords:** *Lilium davidii* var. *unicolor*, hybrid population, heterosis, tepal spots, flower color

## Abstract

*Lilium davidii* var. *unicolor* is an important genetic resource for the origin of Asiatic hybrid lilies and a vital edible lily resource in China. To develop new lily germplasm combining ornamental and edible values, this study conducted five hybrid combinations between *Lilium davidii* var. *unicolor* (abbreviated as LDU) and Tiger/Pearl series Asiatic hybrid cultivars. Fourteen quantitative traits, along with spot patterns and flower color, were measured in 196 individual plants from the hybrid population, encompassing plant growth and ornamental traits. The brightness (L*), red–green component (a*), and yellow–blue component (b*) of flower color were measured and analyzed. Additionally, the genetic variation in growth and ornamental traits among the hybrid progeny was investigated. Studies have shown that the progeny of *Lilium davidii* var. *unicolor* and hybrids with lilies of different ploidy levels exhibit significant diversity in growth traits. Specifically, the F1 generation is characterized by increased plant height and larger flower diameter. Regarding tepal spotting, all five combinations produced both spotted and non-spotted individuals, with a ratio ranging from 3:1 to 5:1. Notable variation in spot distribution and density was observed among spotted individuals, with four combinations exhibiting apparent heterosis, particularly in two combinations involving tetraploid parents. Spots displayed diverse patterns, including scattered, concentrated, and ring-shaped distributions. Cluster analysis based on brightness (L*), redness–greenness (a*), and yellowness–blueness (b*) values categorized the flower colors of the hybrid population into four major types: orange, yellow/yellow-white, light pink, and red. Notably, the hybrids predominantly exhibited enhanced brightness (L*) and yellowness–blueness (b*), with the orange color spectrum being the most prevalent. This study provides a theoretical foundation and practical guidance for the improvement of ornamental traits and germplasm innovation in lilies.

## 1. Introduction

The genus *Lilium* of the family Liliaceae includes approximately 123 recognized species and is widely distributed across the Northern Hemisphere in Asia, Europe, and North America. China, in particular, hosts approximately 55 distinct species according to the latest flora records [1]. Among them, *Lilium davidii* var. *unicolor* (2n = 2x = 24), known as the Lanzhou lily, is a famous edible variety in China with a cultivation history of over 400 years. It is characterized by thick and tender white flesh, a sweet taste, bright flower colors, and recurved floral morphology, making it an excellent germplasm resource with edible, medicinal, and ornamental values [2]. However, *Lilium davidii* var. *unicolor* thrives in cool and temperate environments, which limits its cultivation to specific regions [3].

Research on the ploidy relationships and ploidy inheritance of lilies is a crucial aspect of breeding work. Most wild lilies commonly have 24 chromosomes, representing a diploid state (2n = 2x = 24), with the exception of *Lilium lancifolium*, which exhibits both diploid and triploid forms (2n = 3x = 36). In modern cultivated varieties, ploidy levels vary widely, including diploids, triploids, tetraploids (2n = 4x = 48), and aneuploids. Additionally, some lily chromosomes display the presence of B chromosomes in their karyotypes. Among hybrid Asian lilies, most are diploids, but through modern breeding techniques, triploid, tetraploid, and other polyploid varieties have been developed [4]. Huhao determined the karyotypes of several hybrid Asian lilies through conventional chromosome squashing and karyotype analysis, including ‘Pink Flavour’, ‘Sweet Surrender’, and ‘White Twinkle’ (2n = 2x = 24); ‘Tiger Babies’ and ‘Red Velvet’ (2n = 3x = 36); ‘Pearl Carolina’, ‘Pearl Melanie’, ‘Pearl Loraine’, ‘Red Life’, ‘Pearl Stacey’, ‘Pearl Justin’, and ‘Pink Flight’ (2n = 4x = 48) [5].

In hybrid breeding, ornamental lilies have developed rapidly, while research on edible lily hybrid breeding remains in its early stages [6]. Heterosis, an important approach to enhancing crop quality, yield, and resistance, has been widely applied in crop breeding [7]. The Tiger/Pearl series lilies are common Asiatic hybrid cultivars characterized by diverse flower colors, strong resistance, and short growth cycles [8,9]. Crossbreeding these varieties with *Lilium davidii* var. *unicolor* holds potential for creating new germplasms that combine ornamental and edible traits. Improving the growth and ornamental traits of *Lilium davidii* var. *unicolor* through hybrid breeding is of significant importance [10].

Flower color and tepal spots are crucial ornamental traits in lilies [11]. Understanding their genetic mechanisms is vital for guiding parental selection and hybrid design. The orange color of *Lilium davidii* var. *unicolor* is attributed to a single type of carotenoid pigment [12]. Few studies have reported on flower color traits in the hybrid progeny of *Lilium davidii* var. *unicolor*. Wu et al. [6] crossed white wild lilies with *Lilium davidii* var. *unicolor* and obtained pale-yellow hybrid progeny. Hu Weirong [13] used *Lilium davidii* var. *unicolor* as the maternal parent and various ornamental lilies as paternal parents to study cross-compatibility, discovering good compatibility between *Lilium davidii* var. *unicolor* and the pink Asiatic lily cultivar ‘Pink Flavour’. Wang Zhiyi [4] utilized the superior hybrid progeny of ornamental lilies such as ‘Red Chili’ as maternal parents and crossed them with seven different Asiatic lilies, generating progeny through embryo rescue techniques. These studies indicate efforts in hybrid breeding with *Lilium davidii* var. *unicolor*, but research on flower color traits in its hybrid progeny remains limited. 

Regarding growth traits, the breeding of Asiatic lilies involves complex processes and ploidy variations. Polyploid lilies often exhibit growth advantages such as thicker stems and broader leaves [9,14,15]. Breeding using Tiger/Pearl series lilies with diverse ploidy levels and plant heights may help overcome the slow growth of *Lilium davidii* var. *unicolor* and improve its growth traits.

Currently, the breeding of lily varieties in the market primarily focuses on ornamental traits, such as flower color, shape, fragrance, and plant architecture. In contrast, research on the breeding of edible lilies has stagnated, and to date, no stable and commercially valuable edible varieties have been successfully developed. In recent years, studies on edible lilies have mainly concentrated on cultivation techniques, high-yield and pollution-free production methods, germplasm resources, and growth and development. However, limited progress has been made in breeding research. Furthermore, establishing systematic breeding and cultivation strategies based on the growth characteristics of edible lilies remains crucial [4].

Therefore, this study aimed to hybridize ornamental Tiger/Pearl series lilies (2n = 2x = 24/2n = 3x = 36/2n = 4x = 48) with superior flower diameters, flower colors, plant heights, and number of spots with *Lilium davidii* var. *unicolor* (2n = 2x = 24). Moreover, the Tiger series of hybrid Asian lilies has the genetic background of *Lilium davidii*, which also has edible value [16]. By investigating growth and ornamental traits in the hybrid progeny, this research systematically analyzes genetic variation patterns, providing breeding materials and theoretical support for developing dual-purpose edible and ornamental lily varieties.

## 2. Materials and Methods

**Figure 1:**
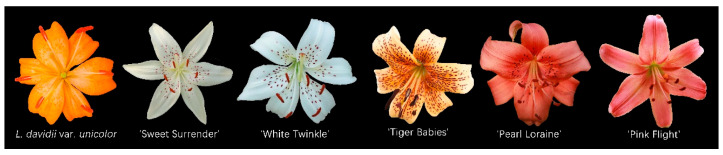
Phenotypes of different parental flowers.

*Lilium davidii* var. *unicolor* (abbreviated as LDU) was selected as one parent and hybridized with Asiatic hybrid lilies from the Tiger/Pearl series, including ‘Sweet Surrender’, ‘White Twinkle’, ‘Tiger Babies’, ‘Pearl Loraine’, and ‘Pink Flight’. The floral characteristics and ploidy levels of the parental lilies are shown in Figure 1 and Table 1.

### 2.1. Design of Hybrid Combinations and Acquisition of Hybrid Populations

Hybridization experiments were conducted in May and June 2020. Crosses were designed using LDU and materials with varying ploidy levels as parents. After artificial pollination, seed sowing, transplantation, and seedling cultivation, the hybrids entered the flowering stage in 2023. The hybrid populations were cultivated in the unheated greenhouse at the Beijing Green Garden Group Plant Center in Shunyi District, Beijing. The traits of individual flowering plants were investigated in June 2024. The number of progeny from each hybrid combination is shown in Table 1.

### 2.2. Measurement of Quantitative Traits in Hybrid Population

In June 2024, phenotypic data were collected for five randomly selected plants from each parental line and for all F1 hybrids. The data were divided into growth traits and floral traits. Growth traits included the following: plant height, stem diameter, leaf length, leaf width, number of flowers. Floral traits included the following: flower diameter, outer perianth length, outer perianth width, inner perianth length, inner perianth width, ovary length, ovary width, stylus length, filament length. For plants with visible tepal spots, the number of spots on the inner and outer tepals was counted separately and then summed to obtain the total number of spots. Measurements followed the trait standards outlined in the UPOV Guidelines for the Conduct of Tests for Distinctness, Uniformity, and Stability of *Lilium* (Republic of China Ministry of Agriculture, 2013). Length-related traits were measured using tape measures and calipers, and countable traits were visually assessed and recorded. Specific measurement standards are summarized (Table 2).

### 2.3. Measurement of Flower Color Traits

A Nippon Denshoku NF555 portable spectrophotometer was used to measure the L*, a*, and b* values of flower color at the center of the inner tepals for each progeny plant. Each measurement was repeated three times per plant, and the mean value was recorded as the final result.

L*, a*, b* represent the three main color dimensions in the color space, defined by the International Commission on Illumination (CIE), and are used to describe the properties of color. Specifically, these values form the CIE LAB color space (commonly referred to as the LAB color space), which is designed based on the human eye’s perception of color and is widely applied in color measurement, display devices, printing, and image processing.

The three dimensions of the CIE LAB color space are as follows:

L* (Lightness): This value represents the degree of lightness or darkness of a color, ranging from 0 (black) to 100 (white). A higher L value indicates a brighter color, closer to white, while a lower L* value indicates a darker color, closer to black.

a* (Green–Red Axis): This value indicates the degree of color shift between green and red. Positive a values indicate a redder hue, while negative a* values indicate a greener hue. Larger positive a* values correspond to a redder color, while smaller a* values correspond to a greener color.

b* (Blue–Yellow Axis): This value indicates the degree of color shift between blue and yellow. Positive b values indicate a yellower hue, while negative b* values indicate a bluer hue. Larger positive b* values correspond to a yellower color, while smaller b* values correspond to a bluer color.

### 2.4. Data Statistics and Analysis

The trait data were recorded and organized using Microsoft Office Excel. Statistical analysis for maximum, minimum, mean, standard deviation, variance, kurtosis, skewness, and Kolmogorov–Smirnov (K-S) normality tests were performed using IBM SPSS Statistics 25 (Version Number: 25.0.0.0). The coefficient of variation (CV), mid-parent values (MPs), high-parent values (HPs), heterosis index (Hi), mid-parent heterosis (Hm), and over-parent heterosis (Ho) were calculated using the following formulas:

$$CV=\frac{\sigma }{\bar{X}}\times 100\%$$

$$MPs=\frac{P1+P2}{2}$$

$$Hi=\frac{\bar{X}}{MPs}\times 100\%$$

$$Hm=\frac{\bar{X}-MPs}{MPs}\times 100\%$$

$$Ho=\frac{\bar{X}-HPs}{HPs}$$

where *P*1 and *P*2 represent the values of the parents, $\bar{X}$ represents the mean value of the hybrids, and *σ* is the standard deviation. Correlation analysis and heatmap visualization were performed using Origin 2021 software. Flower color data for the F1 hybrid population were subjected to cluster analysis, with results visualized through scatterplots, boxplots, and other graphical representations.

## 3. Results

### 3.1. Analysis of Variation and Correlations in Traits Associated with Plant Growth

Fourteen quantitative traits, including plant height, stem diameter, leaf length, leaf width, number of flowers, flower diameter, outer perianth length, outer perianth width, inner perianth length, inner perianth width, ovary length, ovary width, style length, and filament length, were subjected to basic statistical description, kurtosis and skewness calculations, and Kolmogorov–Smirnov (K-S) normality tests (Table 3).

Among these traits, plant height, leaf length, flower diameter, outer perianth length, outer perianth width, inner perianth length, inner perianth width, style length, and filament length exhibited *p*-values greater than 0.05, indicating normal distributions. In contrast, stem diameter, leaf width, number of flowers, ovary length, and ovary width displayed positively skewed distributions.

The coefficients of variation (CVs) ranged from 0.46% to 64.21%, with the number of flowers showing the highest variation (64.21%), followed by stem diameter (41.42%). Growth traits such as plant height, stem diameter, leaf length, and leaf width, which are closely associated with vegetative growth, exhibited significantly higher variation than floral traits. This indicates that floral quantitative traits are genetically more stable.

**Figure 2:**
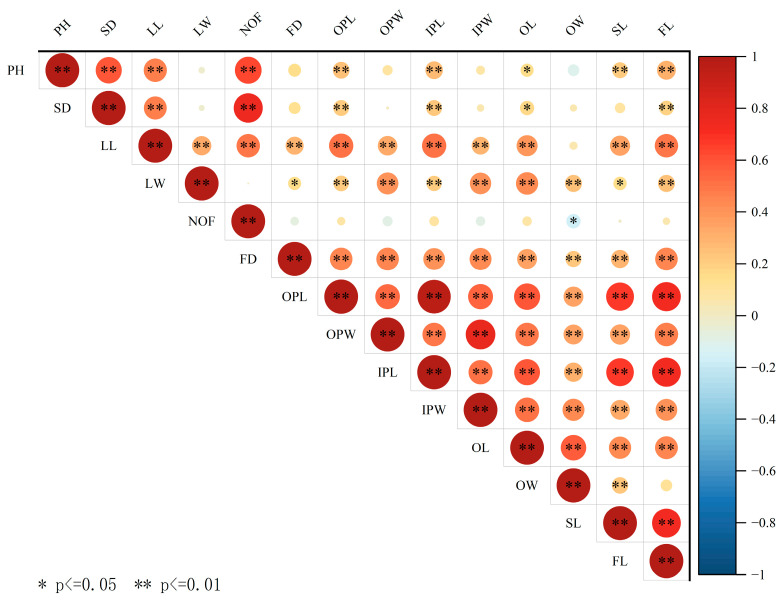
Correlation heatmap of quantitative traits in the F1 hybrid population. Red: positive correlation; deeper shades indicate stronger correlations. Blue: negative correlation; deeper shades indicate stronger correlation significance. Levels: *p* ≤ 0.05 (*), *p* ≤ 0.01 (**).

A correlation analysis was conducted for the quantitative traits of the F1 hybrid population, and the results are visualized in the heatmap (Figure 2); detailed correlation coefficients are provided in Supplementary Materials (Figure S1).

The heatmap reveals that plant height showed a significant positive correlation with stem diameter, leaf length, and the number of flowers, with correlation coefficients of 0.59 and 0.62 for stem diameter and number of flowers, respectively. Similarly, stem diameter exhibited a strong positive correlation with leaf length and the number of flowers, with a coefficient of 0.75 for the latter. These findings indicate that vegetative traits such as plant height and stem diameter are closely associated with reproductive traits such as flower number. However, plant height and stem diameter had minimal correlation with flower diameter, suggesting that the size of the flower is primarily influenced by genetic factors and remains relatively stable.

Flower diameter showed significant positive correlations with floral traits, including the lengths and widths of the inner and outer tepals, ovary, style, and filaments, with correlation coefficients ranging from 0.22 to 0.45.

Based on these analyses, traits such as plant height and stem diameter are strongly linked to flower number, whereas flower diameter is more associated with other floral traits. Therefore, subsequent analyses focused on variations in plant height, stem diameter, and flower diameter across hybrid combinations and their heterosis effects.

### 3.2. Distribution Characteristics and Heterosis Analysis of Plant Height, Stem Diameter, and Flower Diameter in Different Hybrid Combinations

**Figure 3:**
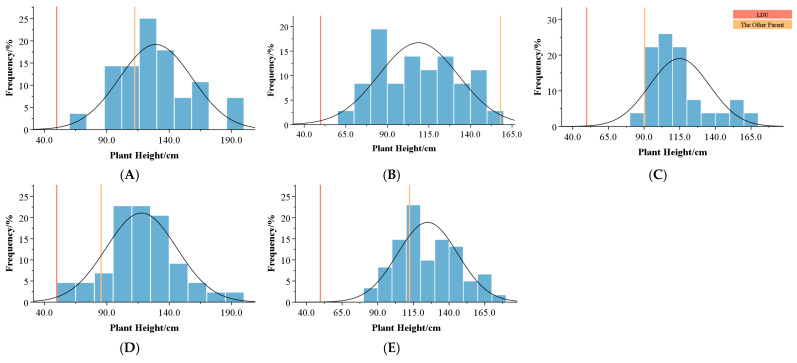
Frequency distribution of plant heights in the F1 hybrids of different combinations. (**A**–**E**) represent different combinations. The vertical orange line represents the value for *Lilium davidii* var. *unicolor*, and the pale orange line represents the value for the other parent.

The distribution and variation in plant height among the progeny of the five hybrid combinations were analyzed (Table 4, Figure 3).

*Lilium davidii* var. *unicolor* has a relatively short plant height. In all hybrid combinations, the height of the other parent exceeded that of *Lilium davidii* var. *unicolor*, and the F1 hybrids demonstrated plant heights greater than those of *Lilium davidii* var. *unicolor*. In combination B (‘White Twinkle’ × LDU), where the maternal parent ‘White Twinkle’ was significantly taller, the F1 hybrid heights fell between the two parents. In the remaining combinations, the proportions of F1 plants taller than the higher parent were 75.0%, 96.3%, 88.64%, and 70.49%, respectively. Corresponding heterosis indices (Hi) were 159.47%, 163.82%, 174.13%, and 154.19%, while over-parent heterosis values (Ho) were 15.09%, 27.04%, 37.82%, and 11.29%. Combination D (LDU × ‘Pearl Loraine’) involving a tetraploid parent exhibited the highest coefficient of variation (24.11%), the highest heterosis index (174.13%), and the highest over-parent heterosis (37.82%).

**Figure 4:**
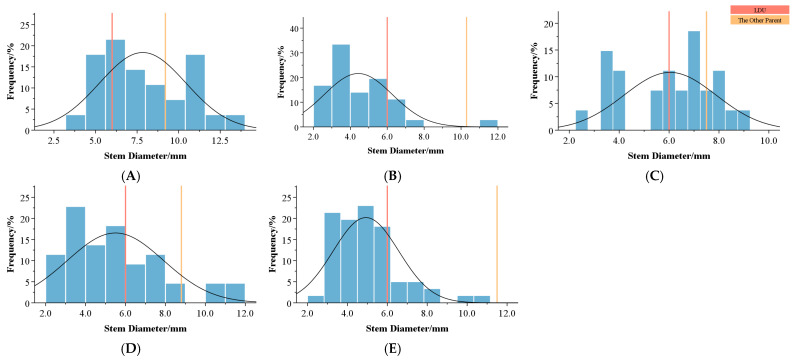
Frequency distribution of stem diameters in the F1 hybrids of different combinations. (**A**–**E**) represent different combinations. The vertical orange line represents the value for *Lilium davidii* var. *unicolor*, and the pale orange line represents the value for the other parent.

For stem diameter (Table 5, Figure 4), all combinations exhibited high coefficients of variation, but no clear patterns were observed among ploidy levels. In combinations A and C, 32.27% and 22.22% of F1 plants exceeded the higher parent value. In the other combinations, most F1 plants had stem diameters between the parental values or smaller than the lower parent. This is likely due to the young age of the progeny and their second-year flowering. Notably, combination A exhibited a positive over-parent heterosis (Ho = 3.16%), indicating superior stem diameter performance in its progeny.

**Figure 5:**
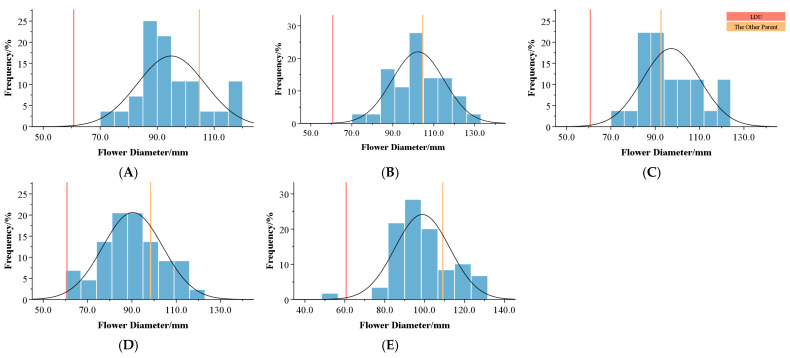
Frequency distribution of flower diameters in the F1 hybrids of different combinations. (**A**–**E**) represent different combinations. The vertical orange line represents the value for *Lilium davidii* var. *unicolor*, and the pale orange line represents the value for the other parent.

Regarding flower diameter (Table 6, Figure 5), *Lilium davidii* var. *unicolor* has a relatively small mean flower diameter of 60.70 mm. Crossing *Lilium davidii* var. *unicolor* with Tiger/Pearl series lilies resulted in significant increases in mean flower diameter across all combinations. Notably, combinations C and D exhibited over-parent heterosis values of 4.99% and 8.14%, respectively.

### 3.3. Genetic Patterns of Tepal Spotting in Different Hybrid Combinations

The presence or absence of tepal spots, the number of spots, distribution patterns, and their frequency distributions were analyzed for the parental lines and hybrid progeny of each combination.

*Lilium davidii* var. *unicolor* lacks tepal spots, presenting only small papillae similar in color to the tepals. Among the other four parental lines hybridized with *Lilium davidii* var. *unicolor*, varying degrees of spotting were observed. For example, ‘Tiger Babies’ (combination C) exhibited the highest number of spots, covering almost the entire tepal surface, whereas ‘Pink Flight’ (combination E) had the fewest, with an average of only 21.84 spots.

In the hybrid progeny, individuals with and without spots were observed in all combinations (Table 7). Spotted individuals outnumbered those without spots across combinations. Both diploid × diploid and diploid × tetraploid combinations displayed segregation ratios of 3:1 or 6:1, while the triploid × diploid combination exhibited a ratio of 1.5:1. These results suggest that the presence of tepal spots is a dominant trait.

**Figure 6:**
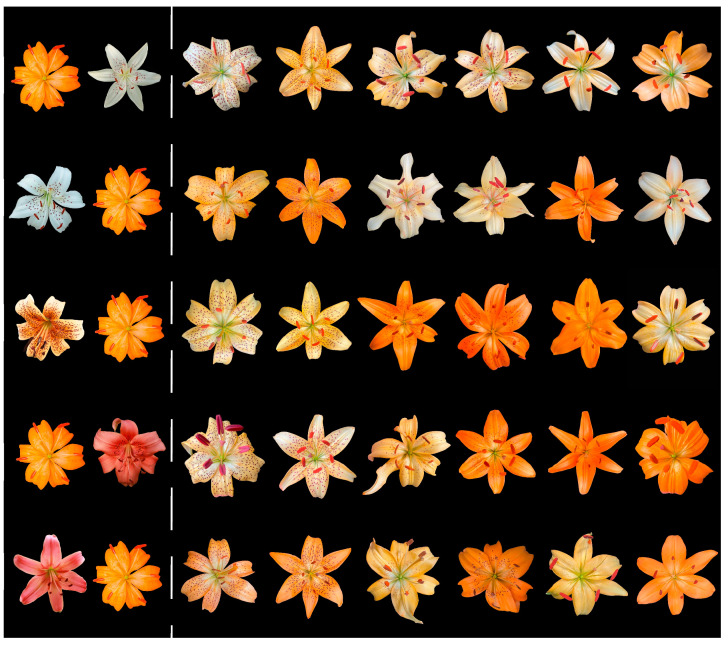
Representative flower images showing tepal spotting patterns. From top to bottom: combinations A, B, C, D, and E in the left four columns represent spotted individuals, while the right two columns represent non-spotted individuals.

Three distinct distribution patterns of tepal spots were observed among the progeny (Figure 6): spots scattered across the entire surface of both inner and outer tepals; spots concentrated on the middle to lower parts of the tepals; and spots forming a near-circular pattern in the middle region of the tepals. 

**Figure 7:**
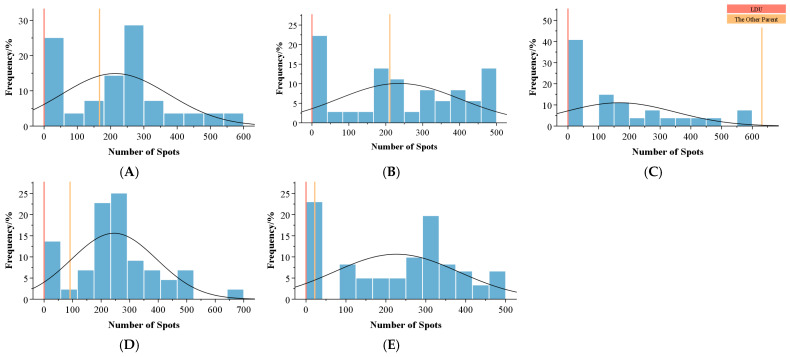
Frequency distribution of the number of spots in the F1 hybrids of different combinations. (**A**–**E**) represent different combinations. The vertical orange line represents the value for *Lilium davidii* var. *unicolor*, and the pale orange line represents the value for the other parent.

Statistical analysis of spot count variation and distribution patterns in the progeny revealed significant variability (Table 8, Figure 7). The coefficients of variation ranged from 58.68% to 108.35%. Combination C (LDU × ‘Tiger Babies’) showed the highest coefficient of variation (108.35%). The frequency distribution of spot counts generally followed a positively skewed or normal distribution.

For combination C, the parental line ‘Tiger Babies’ displayed an extensive number of spots across the tepals, but its progeny exhibited fewer spots. In contrast, in the other four combinations (A, B, D, and E), the proportion of progeny with more spots than the higher parent exceeded 50%. Particularly in the tetraploid combinations D and E, the heterosis index (Hi) values were 558.98% and 2073.08%, respectively, with over-parent heterosis (Ho) values of 458.98% and 1973.08%. In combination E, 86.36% of progeny individuals displayed higher spot counts than the higher parent. These results indicate that the hybrid progeny exhibit rich variation in spot numbers, with an overall trend toward increased spotting. Combinations D and E demonstrated the most pronounced heterosis effects.

### 3.4. Variations in Flower Color

**Figure 8:**
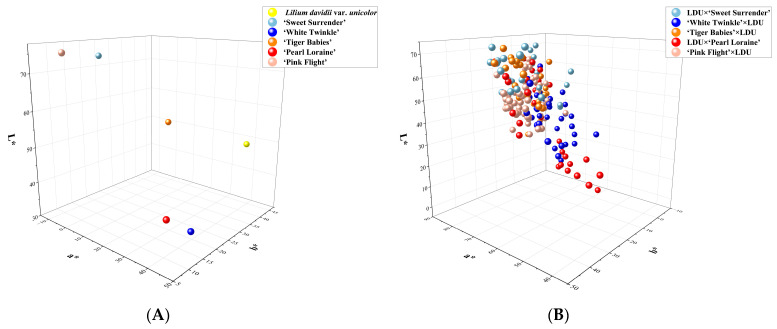
Three-dimensional scatterplots of L*, a*, and b* values in parents (**A**) and F1 progeny (**B**). (Panel **A**) represents the parental L*, a*, and b* values, and (panel **B**) represents the F1 progeny values.

Flower color variations were observed across all five hybrid combinations. The L*, a*, and b* values of parental and hybrid progeny flower colors were measured using a spectrophotometer. A three-dimensional scatterplot was generated to visualize the distribution of these values (Figure 8). Parental lines exhibited significant differences in L*, a*, and b* values. For instance, ‘Sweet Surrender’ and ‘Pink Flight’ showed markedly higher brightness (L*) than the other parents, while their redness (a*) values were significantly lower. The yellowness (b*) value of *Lilium davidii* var. *unicolor* was notably higher than that of the other parents (Figure 8A). The hybrid progeny exhibited considerable variation in flower color distribution and the distribution is more concentrated in regions with higher brightness (L*) and yellow–blue component (b*) values (Figure 8B).

Statistical analysis of the progeny (Table 9) revealed that the mean L* values of all hybrid combinations exceeded the mid-parent values, indicating increased brightness in the progeny. The a* values were lower than the mid-parent values in all combinations except combination A. Meanwhile, the b* values were consistently higher than the mid-parent values. These results suggest that crossing *Lilium davidii* var. *unicolor* with Asiatic hybrid lilies can significantly enhance brightness and yellowness in progeny flower colors, with orange-yellow hues showing strong heritability.

Among the hybrid combinations, the L* values were relatively consistent across combinations and normally distributed. However, significant variation was observed in a* and b* values. For instance, combinations A, C, and E showed greater dispersion in a* values, while combination D exhibited greater variation in b* values, showing a bimodal distribution.

**Table 9:** Statistical indicators of L*, a*, and b* values in the hybrid progeny.

	Cross Combination	Parents			F1				
		♀	♂	MP_s_	Mean	Standard Deviation	Min	Max	CV/%
L*	A	49.39	73.59	61.49	70.93	8.67	53.89	83.49	12.22
	B	75.64	49.39	62.52	72.04	8.15	55.52	85.30	11.31
	C	55.82	49.39	52.61	71.80	9.18	56.11	86.74	12.79
	D	49.39	33.77	41.58	68.82	10.20	42.35	85.43	14.82
	E	37.87	49.39	43.63	73.21	7.18	57.30	84.65	9.81
a*	A	40.53	−6.50	17.02	23.25	16.07	1.46	44.84	69.12
	B	−2.71	40.53	18.91	9.69	10.02	−1.35	33.99	103.41
	C	17.41	40.53	28.97	21.65	15.12	1.39	45.36	69.84
	D	40.53	42.04	41.29	21.18	13.10	1.93	39.61	61.85
	E	37.30	40.53	38.92	20.91	12.58	−2.45	41.02	60.16
b*	A	42.29	21.28	31.79	60.55	8.92	41.49	74.91	14.73
	B	8.18	42.29	25.24	37.89	10.62	12.16	60.67	28.03
	C	29.52	42.29	35.91	55.60	12.67	23.59	73.34	22.79
	D	42.29	17.72	30.01	45.01	18.60	4.90	67.02	41.32
	E	13.00	42.29	27.65	48.14	7.94	29.90	64.57	16.49

**Figure 9:**
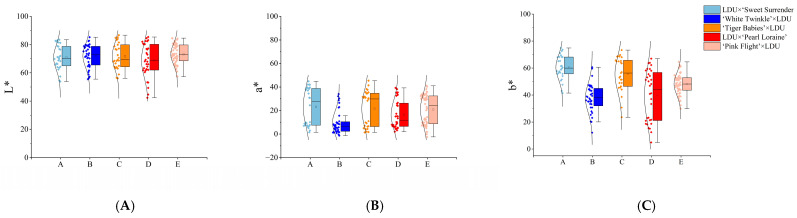
Boxplots of L*, a*, and b* values across hybrid combinations. (**A**–**C**) represent L*, a*, and b* values. A–E on the x-axis represent different hybrid combinations.

Across hybrid combinations, the L* values showed relatively consistent patterns with near-normal distributions. However, substantial variations were observed in the a* and b* values. For example, combinations A, C, and E exhibited greater dispersion in a* values, while combination D displayed notable variability in b* values, with a bimodal distribution trend (Figure 9).

**Figure 10:**
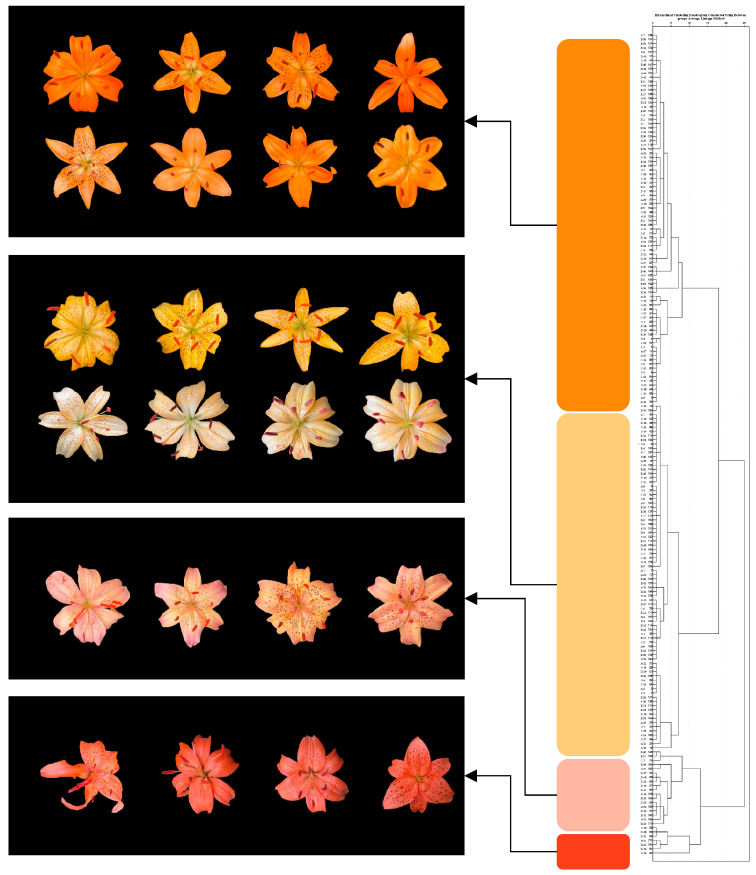
Classification of flower colors in the hybrid progeny. **Right**: cluster dendrogram based on flower color data. **Left**: representative flower images arranged by color groups (from top to bottom: orange, yellow/yellow-white, light pink, and red).

Using L*, a*, and b* values, the hybrid progeny were grouped into four categories through cluster analysis (Figure 10), corresponding to orange, yellow/yellow-white, light pink, and red flower colors. The groups contained eighty-eight (44.84%), eighty-two (42.27%), eighteen (9.28%), and seven (3.61%) individuals, respectively (Table 10).

In the hybrid progeny, regardless of whether the parental lines were diploid, triploid, or tetraploid, individuals with orange or orange-yellow flower colors constituted the majority (87.11% in total), comprising orange and yellow/yellow-white color groups. In the hybrid combinations involving white diploid parental lines, no progeny exhibited flower colors identical to the white parent, whether the white parent served as the maternal or paternal line. Instead, the tepals of the progeny showed varying degrees of carotenoid pigmentation. This indicates that the orange pigmentation of *Lilium davidii* var. *unicolor* has strong heritability, with orange acting as a dominant trait and white as a recessive trait in the inheritance process.

**Figure 11:**
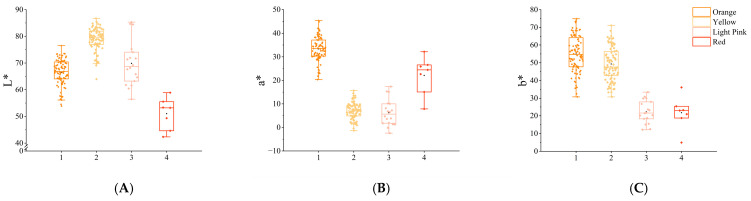
The boxplot of the distribution range of L*, a*, and b* values for different clusters. (**A**–**C**) represent L*, a*, and b* values. Numbers 1 to 4 on the x-axis represent different color clusters.

When red-flowered Asiatic lilies were selected as parental lines for crossing with *Lilium davidii* var. *unicolor*, the resulting progeny exhibited greater color variation. The distribution ranges of L*, a*, and b* values in each cluster were analyzed (Table 11), and boxplots were generated (Figure 11). The results showed that Group 2 had the highest L* values, indicating the greatest brightness. Group 1 exhibited the highest a* and b* values, corresponding to the highest redness and yellowness, respectively. Groups 2 and 3 displayed lower a* values, with significantly reduced redness compared to other groups, while Groups 3 and 4 exhibited lower b* values, indicating reduced yellowness compared to other groups.

## 4. Discussion

Interspecific hybridization is a primary approach for creating new lily varieties. Understanding the genetic mechanisms underlying ornamental and growth traits allows for the rational selection of parental lines, significantly reducing time and economic costs in breeding programs. In this study, *Lilium davidii* var. *unicolor*, a notable dual-purpose edible and ornamental lily germplasm from Gansu Province, China, was used as the breeding material. By constructing hybrid populations with five Asiatic hybrid lilies from the Tiger/Pearl series, we measured 15 key growth and ornamental traits in the progeny and systematically analyzed the genetic variation patterns of these traits.

In lily hybrids, traits such as plant height, stem diameter, and flower number often exhibit substantial heterosis [17,18]. The offspring population derived from *Lilium davidii* var. *unicolor* as the paternal parent and the Asian lily ‘Renoir’ exhibited a high degree of variation in flower number and plant height, with a highly significant positive correlation between these traits. Previous studies have hybridized *Lilium lancifolium* (2n = 3x = 36) with ‘Brunello’ (2n = 4x = 48), and the resulting offspring exhibited a negative mid-parent heterosis of −27.97% for plant height. This suggests that the hybrid progeny could serve as a breeding population for selecting compact varieties. Moreover, the negative heterosis for plant height is of significant value for dwarfing breeding and selecting varieties with improved lodging resistance in lilies [19].

When *Lilium davidii* var. *unicolor* was used as the maternal parent and hybridized with the Asian lily ‘Pink Flavour’ (2n = 2x = 24), the resulting progeny showed higher nutritional composition indicators than the paternal parent, while traits such as leaf width and leaf spacing exhibited intermediate variation [13]. Additionally, chromosome-doubled lilies often exhibit growth characteristics such as thicker stems, larger leaves, and enhanced photosynthetic capacity [20,21]. No studies have reported on the traits of offspring derived from hybridization between *Lilium davidii* var. *unicolor* and polyploid lilies. Due to the limited literature on *Lilium davidii* var. *unicolor* hybridization, breeders face challenges in understanding the genetic patterns associated with its hybridization with polyploid and multi-origin germplasm.

In this study, the plant height of F1 progeny in all five hybrid combinations exceeded that of *Lilium davidii* var. *unicolor*, with four combinations exhibiting high-parent heterosis. The hybrid combination of *Lilium davidii* var. *unicolor* and tetraploid ‘Pearl Loraine’ demonstrated the highest heterosis index, indicating that sexual polyploidization through crosses with higher-ploidy parents positively influences plant height. However, the tallest parent, the diploid ‘White Twinkle’, also produced the tallest F1 progeny, suggesting that parental plant height has a significant impact on progeny performance.

For flower diameter, the mid-parent heterosis was generally high across combinations, with combinations C and D exhibiting over-parent heterosis. These findings suggest that hybridization can significantly improve flower size.

Tepal spotting is an important ornamental trait in lilies. Among the five hybrid combinations between spotless *Lilium davidii* var. *unicolor* and spotted Asiatic hybrids, a small number of spotless progeny were observed. This contrasts with the findings for the hybrid combination of ‘Renoir’ (with sparse spotting) and *Lilium davidii* var. *unicolor*, where all progeny exhibited spots [21]. Previous studies [11,22] have shown that the presence or absence of raised tepal spots is controlled by a single locus. The results of this study suggest that spotted tepals are a dominant trait. However, whether this is governed by a single gene requires further validation in larger populations.

The number of spots in the hybrid progeny exhibited extensive variation and continuous segregation, with an overall trend toward increased spotting. In particular, combinations involving diploid and tetraploid parents produced progeny with significantly more spots than the parents, especially in the case of ‘Pink Flight’ (4x) and *Lilium davidii* var. *unicolor*, which yielded numerous highly spotted progeny. Additionally, the diverse spot distribution patterns observed in the progeny provide opportunities for developing varieties with various spot configurations.

Flower color analysis revealed that orange and yellow/yellow-white flowers dominated in the hybrid progeny across all combinations, reflecting the strong heritability of carotenoids in *Lilium davidii* var. *unicolor*. This finding aligns with previous studies [23,24], which reported that crosses between light pink Asiatic lilies (containing anthocyanins) and orange lilies (containing carotenoids) produced predominantly orange progeny. Moreover, when light pink lilies were crossed with red lilies (containing both anthocyanins and carotenoids), the resulting progeny exhibited extensive flower color segregation [23]. Therefore, further backcrossing of *Lilium davidii* var. *unicolor* F1 progeny with red or pink Asiatic lilies is needed to generate hybrids with a broader range of flower colors.

In future hybridization efforts, using bi-color Asiatic lilies containing both anthocyanins and carotenoids as parents could help develop hybrid progeny with even greater color diversity.

In summary, this study provides scientific evidence for the improvement of ornamental traits in lilies, particularly for plant height, flower diameter, and flower color variation. Future research should further explore other important traits in hybrid progeny, such as bulb metabolites and disease resistance, and analyze their correlations with ornamental traits to achieve more precise trait improvement. Additionally, modern biotechnological approaches, such as marker-assisted selection (MAS), can be employed to enhance the efficiency and accuracy of targeted breeding in lilies.

## Figures and Tables

**Table 1:** Ploidy levels of hybrid parents and progeny population sizes.

No.	♀	♂	Number
A	LDU (2x)	‘Sweet Surrender’ (2x)	28
B	‘White Twinkle’ (2x)	LDU (2x)	36
C	‘Tiger Babies’ (3x)	LDU (2x)	27
D	LDU (2x)	‘Pearl Loraine’ (4x)	44
E	‘Pink Flight’ (4x)	LDU (2x)	61
Total			196

**Table 2:** Measurement standards for traits.

No.	Traits	Abbreviation	Measurement Standard
1.	Plant height	PH/cm	Vertical distance from the ground to the inflorescence apex
2.	Stem diameter	SD/mm	Diameter measured 5–15 cm above the ground
3.	Leaf length	LL/cm	Length from the base to the tip of the longest leaf
4.	Leaf width	LW/cm	Maximum width of the longest leaf
5.	Number of flowers	NOF	Maximum number of flowers per plant
6.	Flower diameter	FD/cm	Widest distance across the flower’s radiating symmetry
7.	Outer perianth length	OPL/cm	Central axis length of the outer tepals
8.	Outer perianth width	OPW/cm	Maximum width of the outer tepals
9.	Inner perianth length	IPL/cm	Central axis length of the inner tepals
10.	Inner perianth width	IPW/cm	Maximum width of the inner tepals
11.	Ovary length	OL/cm	Length from the ovary base to its apex
12.	Ovary width	OW/mm	Maximum transverse diameter of the ovary
13.	Style length	SL/cm	Length from the base to the apex of the style
14.	Filament length	FL/cm	Absolute length of the filaments
15.	Number of spots	NOS	Total number of spots on inner and outer tepals

**Table 3:** Descriptive statistics and normality test results.

Traits	Min	Max	Mean	Standard Deviation	CV/%	Skewness	Kurtosis	Kolmogorov–Smirnov Test (*p*)
Plant height	56.20	195.00	119.57	25.33	21.18	0.287	0.203	0.087
Stem diameter	2.20	13.00	5.53	2.29	41.42	0.975	0.558	0.000
Leaf length	67.10	178.50	104.21	18.42	17.67	0.494	0.555	0.200
Leaf width	2.40	9.00	4.77	1.32	27.65	0.789	0.385	0.000
Number of flowers	1.00	14.00	4.62	2.97	64.21	0.815	0.112	0.000
Flower diameter	56.20	130.80	96.80	13.66	14.11	0.145	−0.008	0.200
Outer perianth length	51.40	94.00	71.63	8.35	11.66	−0.002	−0.328	0.200
Outer perianth width	14.60	26.80	21.25	2.25	10.59	0.005	−0.258	0.200
Inner perianth length	49.40	94.20	70.82	8.37	11.81	0.036	−0.228	0.200
Inner perianth width	21.90	39.50	29.49	3.39	11.50	0.492	0.175	0.200
Ovary length	8.30	19.10	12.10	1.85	15.29	0.589	0.403	0.002
Ovary width	2.20	4.70	3.15	0.46	14.56	0.446	0.062	0.001
Style length	25.30	56.80	42.24	5.96	14.11	−0.156	−0.302	0.078
Filament length	32.50	66.00	51.84	4.73	9.13	−0.340	0.970	0.200

**Table 4:** Plant height variation and distribution patterns in the hybrid progeny.

Cross Combination	Parent			F1			Hi/%	Ho/%	Hybrid Ratio/%		
	♀	♂	MPs	$\bar{X}$ ± σ	Range (Min~Max)	CV/%			<LP	Between Parents	>HP
A	49.8	112.3	81.05	129.25 ± 29.13	71.30~195.00	22.68	159.47	15.09	0	25	75
B	158	49.8	103.9	108.71 ± 23.87	65.50~150.90	21.96	104.63	−31.2	0	100	0
C	90.4	49.8	70.1	114.84 ± 20.96	88.90~165.60	18.25	163.82	27.04	0	3.7	96.3
D	49.8	85.42	67.61	117.73 ± 28.38	56.20~187.50	24.11	174.13	37.82	0	11.36	88.64
E	112.29	49.8	81.05	124.97 ± 21.14	85.90~176.30	16.92	154.19	11.29	0	29.51	70.49

$\bar{X}$ represents the mean value of the hybrids, and *σ* is the standard deviation. CV represents the coefficient of variation. Hi represents the heterosis index; Ho represents over-parent heterosis. LP represents the lower parent value and HP represents the higher parent value.

**Table 5:** Stem diameter variation and distribution patterns in the hybrid progeny.

Cross Combination	Parent			F1			Hi/%	Ho/%	Hybrid Ratio/%		
	♀	♂	MPs	$\bar{X}$ ± σ	Range (Min~Max)	CV/%			<LP	Between Parents	>HP
A	6	9.2	7.6	7.84 ± 2.60	3.50~13.00	33.16	103.16	3.16	21.43	46.43	32.27
B	10.3	6	8.2	4.43 ± 1.85	2.20~11.90	41.76	54.02	−45.98	83.33	13.89	2.78
C	7.5	6	6.8	6.04 ± 1.85	2.50~8.90	30.63	88.82	−11.18	40.74	37.04	22.22
D	6	8.8	7.4	5.50 ± 2.41	2.20~11.40	43.82	74.32	−25.68	65.91	22.73	11.36
E	11.5	6	8.8	4.93 ± 1.65	2.80~11.10	33.47	56.02	−43.98	81.97	18.03	0

$\bar{X}$ represents the mean value of the hybrids, and *σ* is the standard deviation. CV represents the coefficient of variation. Hi represents the heterosis index; Ho represents over-parent heterosis. LP represents the lower parent value and HP represents the higher parent value.

**Table 6:** Flower diameter variation and distribution patterns in the hybrid progeny.

Cross Combination	Parent			F1			Hi/%	Ho/%	Hybrid Ratio/%		
	♀	♂	MPs	$\bar{X}$ ± σ	Range (Min~Max)	CV/%			<LP	Between Parents	>HP
A	60.70	104.80	82.75	94.93 ± 11.92	71.20~118.60	12.56	114.72	−9.42	0.00	82.14	17.86
B	104.60	60.70	82.65	102.31 ± 12.70	73.50~130.80	12.41	123.79	−2.19	0.00	61.11	38.89
C	92.60	60.70	76.65	97.22 ± 12.94	75.50~123.70	13.31	126.84	4.99	0.00	48.15	51.85
D	60.70	98.40	79.55	90.39 ± 13.56	64.20~122.90	15.00	113.63	8.14	0.00	72.73	27.27
E	109.10	60.70	84.90	98.87 ± 13.76	56.20~129.00	13.83	116.45	−9.38	1.67	96.00	2.33

$\bar{X}$ represents the mean value of the hybrids, and *σ* is the standard deviation. CV represents the coefficient of variation. Hi represents the heterosis index; Ho represents over-parent heterosis. LP represents the lower parent value and HP represents the higher parent value.

**Table 7:** Segregation of tepal spotting in the hybrid progeny.

Cross Combination	Parent					F1		
	♀	NOS	♂	NOS	With Spots	Without Spots	Proportion	Number
A	LDU (2x)	0.00	‘Sweet Surrender’ (2x)	166.68	21	7	3/1	28
B	‘White Twinkle’ (2x)	210.90	LDU (2x)	0.00	31	5	6/1	36
C	‘Tiger Babies’ (3x)	631.20	LDU (2x)	0.00	16	11	1.5/1	27
D	LDU (2x)	0.00	‘Pearl Loraine’ (4x)	91.02	38	6	6/1	44
E	‘Pink Flight’ (4x)	21.84	LDU (2x)	0.00	47	14	3/1	61

NOS represents the number of spots.

**Table 8:** Number of spots: variation and distribution patterns in the hybrid progeny.

Cross Combination	Parent			F1			Hi/%	Ho/%	Hybrid Ratio/%		
	♀	♂	MPs	$\bar{X}$ ± σ	Range (Min~Max)	CV/%			<LP	Between Parents	>HP
A	0.00	166.68	83.34	213.54 ± 160.62	0.00~555.00	75.22	256.23	156.23	0.00	35.71	64.29
B	210.90	0.00	105.45	236.00 ± 165.05	0.00~492.00	69.94	223.80	123.80	0.00	44.44	55.56
C	631.20	0.00	315.60	167.11 ± 181.07	0.00~564.00	108.35	52.95	−47.05	0.00	100.00	0.00
D	0.00	91.02	45.51	254.39 ± 149.28	0.00~651.00	58.68	558.98	458.98	0.00	13.64	86.36
E	21.84	0.00	10.92	226.38 ± 156.29	0.00~447.00	69.04	2073.08	1973.08	0.00	23.33	76.67

$\bar{X}$ represents the mean value of the hybrids, and *σ* is the standard deviation. CV represents the coefficient of variation. Hi represents the heterosis index; Ho represents over-parent heterosis. LP represents the lower parent value and HP represents the higher parent value.

**Table 10:** Flower color classification in the hybrid progeny.

Cross Combination	Orange	Yellow/Yellow-White	Light Pink	Red
A	15	13	0	0
B	5	21	8	2
C	15	11	1	0
D	20	12	7	5
E	33	25	2	0
Total	88	82	18	7

**Table 11:** Distribution ranges of L*, a*, and b* values for different clusters.

Group	*n* (F1)	Per. (%)	L*		a*		b*	
			Range	Mean	Range	Mean	Range	Mean
1	87	44.84	53.89~76.48	66.54	20.32~45.36	33.37	30.75~74.91	54.94
2	82	42.27	64.03~86.74	78.88	−1.35~15.65	6.91	30.74~71.09	49.29
3	18	9.28	56.44~85.29	69.88	−2.45~17.36	6.25	12.16~33.42	22.30
4	7	3.61	42.35~58.86	51.03	7.87~32.15	22.09	4.90~36.09	21.73

## Data Availability

Data are contained within the article.

## Supplementary Materials

The following supporting information can be downloaded at: https://www.mdpi.com/article/10.3390/plants14050656/s1, Figure S1: Correlation coefficients of quantitative traits in F1 hybrid population.
