# Supplementary figures and images for: Genetic Variation in Ornamental and Growth Traits in Hybrid Populations of Lilium davidii var. unicolor
Source: Plants (Basel). 2025 Feb 21;14(5):656. doi: 10.3390/plants14050656 (PMC11902008; doi:10.3390/plants14050656)

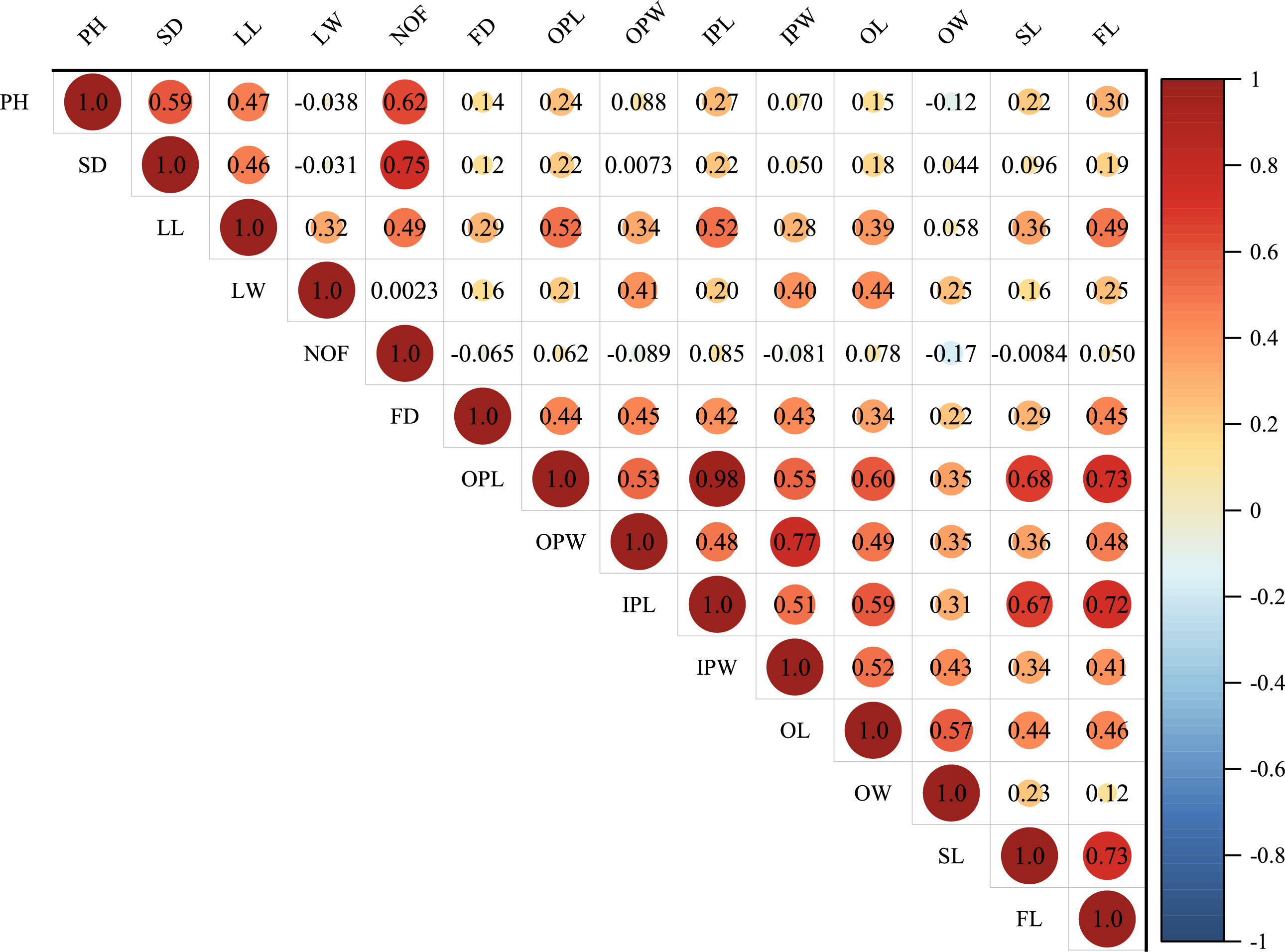

Supplement: Supplementary file 1 [file plants-14-00656-s001.zip › Supplementary/Figure S1.png]
